# The “Oil-Spill Snorkel”: an innovative bioelectrochemical approach to accelerate hydrocarbons biodegradation in marine sediments

**DOI:** 10.3389/fmicb.2015.00881

**Published:** 2015-09-04

**Authors:** Carolina Cruz Viggi, Enrica Presta, Marco Bellagamba, Saulius Kaciulis, Santosh K. Balijepalli, Giulio Zanaroli, Marco Petrangeli Papini, Simona Rossetti, Federico Aulenta

**Affiliations:** ^1^Water Research Institute, National Research CouncilRome, Italy; ^2^Institute for the Study of Nanostructured Materials, National Research CouncilRome, Italy; ^3^Department of Civil, Chemical, Environmental and Materials Engineering, University of BolognaBologna, Italy; ^4^Department of Chemistry, Sapienza University of RomeRome, Italy

**Keywords:** anoxic marine sediments, bioelectrochemical systems, crude oil pollution, *in situ* bioremediation, Oil-Spill Snorkel

## Abstract

This study presents the proof-of-concept of the “Oil-Spill Snorkel”: a novel bioelectrochemical approach to stimulate the oxidative biodegradation of petroleum hydrocarbons in sediments. The “Oil-Spill Snorkel” consists of a single conductive material (the snorkel) positioned suitably to create an electrochemical connection between the anoxic zone (the contaminated sediment) and the oxic zone (the overlying O_2_-containing water). The segment of the electrode buried within the sediment plays a role of anode, accepting electrons deriving from the oxidation of contaminants. Electrons flow through the snorkel up to the part exposed to the aerobic environment (the cathode), where they reduce oxygen to form water. Here we report the results of lab-scale microcosms setup with marine sediments and spiked with crude oil. Microcosms containing one or three graphite snorkels and controls (snorkel-free and autoclaved) were monitored for over 400 days. Collectively, the results of this study confirmed that the snorkels accelerate oxidative reactions taking place within the sediment, as documented by a significant 1.7-fold increase (*p* = 0.023, two-tailed *t*-test) in the cumulative oxygen uptake and 1.4-fold increase (*p* = 0.040) in the cumulative CO_2_ evolution in the microcosms containing three snorkels compared to snorkel-free controls. Accordingly, the initial rate of total petroleum hydrocarbons (TPH) degradation was also substantially enhanced. Indeed, while after 200 days of incubation a negligible degradation of TPH was noticed in snorkel-free controls, a significant reduction of 12 ± 1% (*p* = 0.004) and 21 ± 1% (*p* = 0.001) was observed in microcosms containing one and three snorkels, respectively. Although, the “Oil-Spill Snorkel” potentially represents a groundbreaking alternative to more expensive remediation options, further research efforts are needed to clarify factors and conditions affecting the snorkel-driven biodegradation processes and to identify suitable configurations for field applications.

## Introduction

Petroleum hydrocarbons are released into the marine environment both from natural seeps and from anthropogenic activities involved in the drilling, manufacturing, storing, and transporting of crude oil and oil products (Gong et al., 2014).

Application of oil dispersants has been a critical response measure to mitigate impacts of marine oil spill for decades (Prince, 2015). This approach makes the oil spill less visible by reducing the size of oil droplets, changing the oil surface physicochemical properties and increasing oil dispersion in the water column (Gong et al., 2014). Nevertheless, dispersants and dispersed oil under the ocean surface are hazardous for marine life (Kirby and Law, 2008). Particularly, once the dispersed oil reaches the sediments it tends to persist there for a very long time due to the prevailing anoxic conditions which drastically limit the occurrence of oxidative biodegradation processes. Both physical–chemical and biological treatment methods (i.e., bioremediation) have been proposed for the cleanup of oil polluted sediments, with the latter receiving a greater attention due to the lower costs, lower environmental impact, and wide applicability to a range of contamination scenarios (Fodelianakis et al., 2015). So far, a number of strategies have been proposed to enhance the *in situ* biodegradation of petroleum hydrocarbons, including the addition of nutrients, (bio)surfactants, electron acceptors and even oil-degrading bacteria (Nikolopoulou et al., 2013; Singh et al., 2014). The interplay between the (bio)availability of electron acceptors (e.g., oxygen, sulfate, nitrate) and hydrocarbons, as the electron donors and carbon sources for microorganisms, is certainly one of the most important factors influencing the performance of sediment bioremediation systems (Lu et al., 2014a).

Leading to faster rates of hydrocarbons activation and biodegradation, aerobic bioremediation is frequently preferred over its anaerobic counterpart. In this context, a number of engineered approaches have been proposed to effectively deliver oxygen to contaminated sediments. Among them, a modular slurry system which performs *in situ* aeration of the contaminated sediments, while minimizing the risk of spreading the contamination away from the treatment zone, has been recently developed (Genovese et al., 2014). Although the system turned out to be highly effective in stimulating the metabolism of aerobic hydrocarbon-degrading bacteria and in reducing sediment toxicity, its application is highly labor-, and energy-intensive. Other methods include the addition of oxygen-releasing compounds (e.g., calcium peroxide-based chemicals) to the contaminated sediment (Abdallah et al., 2009). However, the rapid abiotic oxygen consumption by reactions with reduced species (e.g., Fe^2+^, S^2-^) and the difficulties in controlling the rate of oxygen release over time make these approaches often poorly effective (Borden et al., 1997).

Recently, bioelectrochemical systems employing solid-state (graphite) electrodes as electron donors or acceptors have been proposed to stimulate biodegradation processes in subsurface environments (Aulenta et al., 2011a,b; Lovley, 2011; Lu et al., 2014b; Li and Yu, 2015). As an example, *Geobacter metallireducens* was shown to be capable of oxidizing toluene with a graphite electrode (polarized at +500 mV vs. the standard hydrogen electrode with a potentiostat) serving as a direct electron acceptor (Zhang et al., 2010). Other recent studies have shown that lab-scale microbial fuel cells, typically using carbon-based anodes and catalyzed cathodes exposed to air, can be used to accelerate the biodegradation of oil hydrocarbons in contaminated soil and sediment (Morris and Jin, 2012; Lu et al., 2014a,b). In principle, the use of electrodes to stimulate the microbiological oxidation of hydrocarbons in subsurface environments is extremely appealing since they can potentially serve as permanent, low-cost, low maintenance source of electron acceptor capacity (Zhang et al., 2010).

**FIGURE 1:**
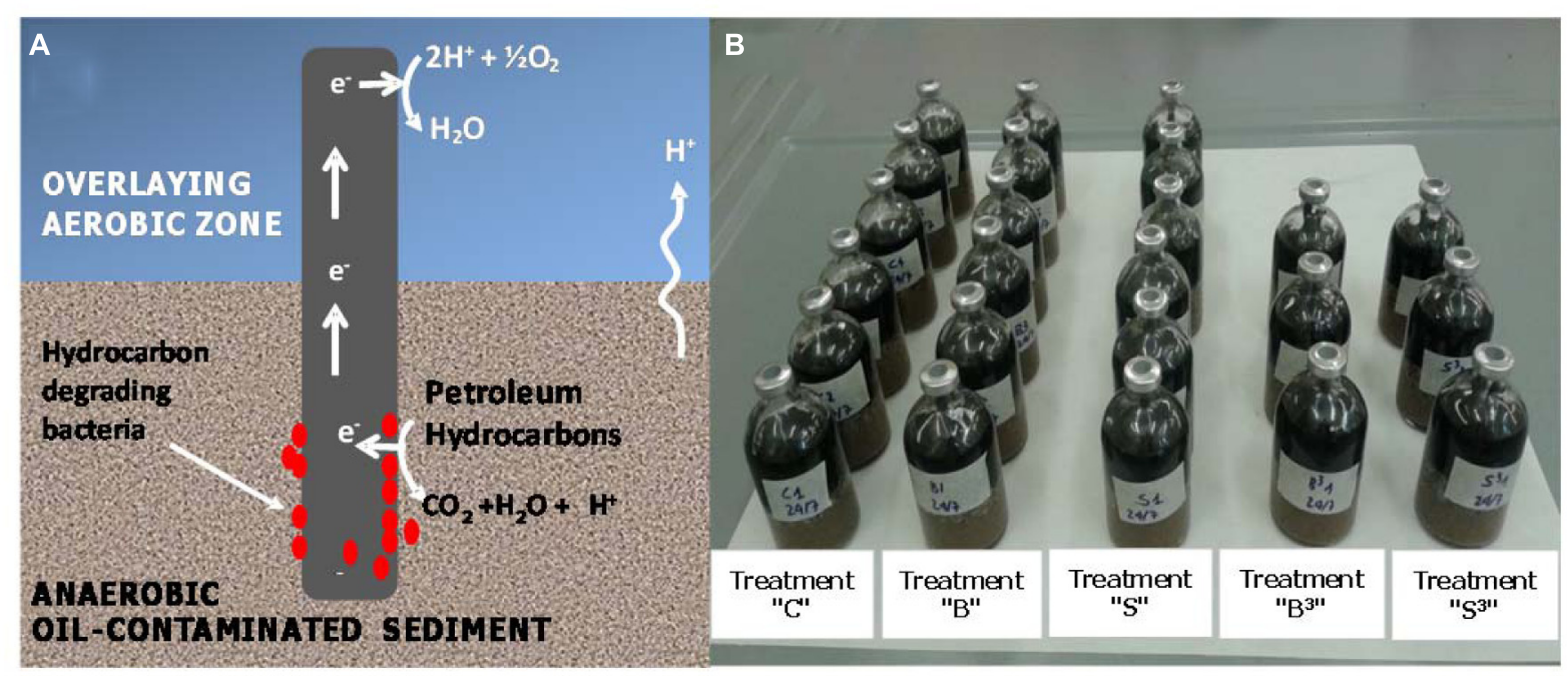
**(A)** Conceptual illustration of the “Oil-Spill Snorkel.” The segment of the electrode that is buried within the sediment serves as an anode, accepting the electrons deriving from the microbiological oxidation of contaminants and other reduced species. Electrons flow through the conductive material up to the portion exposed to the aerobic environment (i.e., the cathode), where they reduce oxygen to form water. **(B)** Picture of the experimental setup.

The aim of this study is to present the proof-of-concept of a novel bioelectrochemical approach to accelerate hydrocarbons biodegradation in anoxic marine sediment. The system, hereafter named “Oil-Spill Snorkel” is based on the “Microbial Electrochemical Snorkel” (MES) concept originally developed for the treatment of urban wastewaters (MFCs; Erable et al., 2011). The “Oil-Spill Snorkel” consists of a single conductive material (i.e., an electrode) positioned so as to create an electrochemical connection between an anoxic zone (e.g., the contaminated sediment) and an oxic zone (i.e., the overlying O_2_-containing water). The lower segment of the electrode, buried within the contaminated sediment, serves as the anode accepting the electrons deriving from the microbiological oxidation of contaminants and other reduced species. Electrons flow through the conductive material to the portion exposed to the aerobic environment (i.e., the cathode) where they reduce oxygen to form water (**Figure 1A**; Erable et al., 2011). In principle, this configuration virtually eliminates the electric resistances resulting from electrode separation in conventional microbial fuel cells and other engineered bioelectrochemical systems. Overall, the “Oil-Spill Snorkel” aims at providing bacteria within the sediment access to a high redox potential electron acceptor (oxygen) and by so doing accelerating oxidative biodegradation processes. To our knowledge, this is the first time that a MES is applied for bioremediation purposes and specifically for the treatment of marine sediments contaminated by petroleum hydrocarbons.

## Materials and Methods

### Experimental Setup

The hereafter described microcosm experiments were carried out using sandy marine sediments from Messina Harbour (Italy; Bargiela et al., 2015). The sediment was artificially contaminated in the laboratory with crude oil (Intermediate Fuel Oil, IFO 180). To this aim, the available sediment was firstly divided into four parts (i.e., quartering); one part was thoroughly mixed with oil that was pre-dissolved in hexane. Then, the hexane was evaporated through the air-drying of the sediment. Finally, the contaminated sediment was mixed, under nitrogen atmosphere, with the remaining three parts of the sediment. This procedure has been used in order to achieve a homogenous contamination of the sediment while preserving a large fraction of the sediment microbial communities.

The so-prepared contaminated sediment was used to setup sacrificial microcosms in 120-mL serum bottles. Each bottle was filled (starting from the bottom) with 50 g of contaminated sediment, 40 g of clean sand, 10 g of Norit granular activated carbon (serving as oxygen reduction catalyst; Zhang et al., 2009, 2011; Watson et al., 2013), and 40 mL of oxygenated seawater from the site. Graphite rods, one or three depending on the treatment (7.5 cm length, 0.6 cm diameter, Sigma–Aldrich, Italy) were also inserted vertically through the layers of the different materials to create the electrochemical connection between the anoxic sediment and the oxygenated overlaying water (**Figures 1A,B**). Five different treatments were setup, namely: treatment “S^3^” which contained three graphite rods, treatment “S” which contained one graphite rod, treatment “C” (biotic control) which contained no graphite rods, treatment “B^3^” (autoclaved control) which contained three graphite rods and was autoclaved (120°C for 1 h) on three successive days and treatment “B” (autoclaved control) which contained one graphite rod and was also autoclaved (120°C for 1 h) on three successive days (**Figure 1B**).

Once prepared all the microcosms were statically incubated in the dark in a temperature-controlled room at 20 ± 1°C. Weekly, the headspace of the bottles was analyzed for oxygen consumption and carbon dioxide evolution by gas-chromatography (GC) with thermal conductivity detector (TCD), as described in the following “Analytical Methods” section. Every time the analyses indicated that oxygen concentration was below 5% (v/v), it was re-added by flushing the headspace with air. At fixed times, one bottle from each treatment was sacrificed: the sediment was analyzed (upon liquid–solid extraction) by GC and flame ionization detector (FID) for quantification of hydrocarbons; the liquid phase was analyzed by ion chromatography (IC) for quantification of seawater anions. The abundance and composition of the biomass in the sediment and attached to the graphite rods was also analyzed by *in situ* hybridization techniques.

### Analytical Methods

Concentration of oxygen and carbon dioxide in the headspace of the microcosms was analyzed by injecting 50 μL of gaseous samples, taken with a gastight syringe (Hamilton, Reno, NV, USA), into a Perkin–Elmer Auto System GC (stationary phase: stainless-steel column packed with molecular sieve (Supelco, USA); carrier gas: N_2_ at 20 mL/min; oven temperature: 225°C; injector temperature: 200°C; thermal conductivity detector (TCD) temperature: 200°C).

The anions in the liquid phase were determined by injecting a filtered sample (0.22 μm porosity) into an IC (IonPac AS14 analytical column, Dionex DX-100 system, Dionex Corp., Sunnyvale, CA, USA).

Quantification of total petroleum hydrocarbons (TPH) in the sediment was performed by GC-FID, upon liquid–solid extraction. In brief, sediment samples (∼5 g) were air dried and extracted with a Dionex ASE 200 (Dionex Corp., Sunnyvale, CA, USA) using an acetone:hexane (1:1 v/v) mixture at 100°C and a system pressure of 1500 psi. The obtained extract was evaporated under nitrogen flow and then re-dissolved in 10 mL of an n-heptane solution containing n-dodecane (n-C_12_) and n-tetracontane (n-C_40_), each at 10 mg/L, as integration markers for the GC analysis. Subsequently, the heptane solution was purified using solid phase extraction cartridges filled with Florisil^®^ and anhydrous sodium sulfate (Chromabond^®^ Na_2_SO_4_/Florisil^®^, 6 mL polypropylene columns, 2g/2g) to remove polar components and non-petroleum compounds.

A sample (1 μL) of the purified solution was injected (in pulsed splitless mode) into a GC-FID (Perkin Elmer Clarus 480; column: HP-5 (Agilent) 30 m, ID 0.25 mm, d_f_ 0.25 μm; carrier gas: helium at 20 psi; injector temperature: 270°C; detector temperature: 320°C; oven temperature program: initial 40°C, hold for 1 min, 10°C/min to 200°C, 20°C/min to 320°C, hold for 10 min).

The TPH amount was determined by summing up both the unresolved and resolved components eluted from the GC capillary column between the retention times of n-C_12_ and of n-C_40_, using solutions of diesel motor oil and diesel mineral oil in hexane as calibration standards (concentration range 0.15–2 g/L).

### Molecular Analysis of the Microbial Communities

Sediment samples and biofilms growing on the surface of the graphite rods (i.e., the snorkels) were taken for microbiological analysis. In brief, approximately 1 g of sediment was immediately fixed in formaldehyde (2% v/v final concentration) and processed to extract cells from sediment particles as elsewhere described (Barra Caracciolo et al., 2005). The detached cells were filtered through 0.2 μm polycarbonate filters (Ø 47 mm, Millipore) by gentle vacuum (<0.2 bar) and stored at –20°C until use. To fix the biomass from the biofilm formed at the graphite rods, the surface of the electrode was gently scraped with a sterile spatula. The detached biomass was initially collected in PBS buffer containing 2% v/v formaldehyde, and then filtered as described above.

Catalyzed Reporter Deposition-Fluorescence *In Situ* Hybridization (CARD-FISH) was carried out following previously published protocol (Fazi et al., 2008) using probes targeting *Bacteria* (EUB338 I,II,III), *Archaea* (ARCH915), *alphaproteobacteria* (ALF968), *betaproteobacteria* (BET42A), *gammaproteobacteria* (GAM42A), *deltaproteobacteria* (DELTA495a,b,c), *Firmicutes* (LGC354a,b,c), *Actinomycetes* (HGC69a), *Cytophaga/Bacteroidetes* (CF319), and *Chloroflexi* (GNSB941 and CFX1223).

Probes, labeled with horseradish peroxidase (HRP), were purchased from BIOMERS^1^. Probe details and conditions are reported in probeBase^2^. DAPI (4′,6-diamidino-2-phenylindole) staining was performed for determining total cell numbers, from which the relative abundances of each targeted bacterial population was calculated. Total cells count was performed at the end of the hybridization assay by using Vectashield Mounting Medium with DAPI (Vector Labs, Italy). At least 20 randomly selected microscopic fields for each sample were analyzed to enumerate the cells by microscopic analysis. Slides were examined by epifluorescence microscopy (Olympus, BX51) and the images were captured with Olympus F-View CCD camera and handled with CellˆF software (Olympus, Germany).

### X-Ray Photoelectron Spectroscopy of Graphite Electrodes

The surface chemical composition of the graphite rods was determined by using the X-ray photoelectron spectroscopy (XPS). XPS measurements were performed in an Escalab 250Xi spectrometer (Thermo Fisher Scientific, UK) equipped with a monochromatic Al Kα source and a 6-channeltron detection system. The photoemission spectra were collected at the base pressure of 5 × 10^-8^ Pa, by using 40 eV pass energy of the analyzer and standard electromagnetic lens mode with about 1 mm diameter of analyzed area. Spectroscopic data were processed by the Avantage v.5 software. Shirley background and mixed Lorentzian/Gaussian peak shape (30%) with linked peak widths were used for the peak fitting.

## Results

### Oxygen Consumption and Carbon Dioxide Evolution

The working hypothesis of the “Oil-Spill Snorkel” is that the graphite electrode (i.e., the snorkel) creates an electrochemical connection between the anoxic sediment and the overlying aerobic seawater and, by so doing, provides sediment microorganisms with the opportunity to “respire” a spatially distant electron acceptor (i.e., oxygen).

**FIGURE 2:**
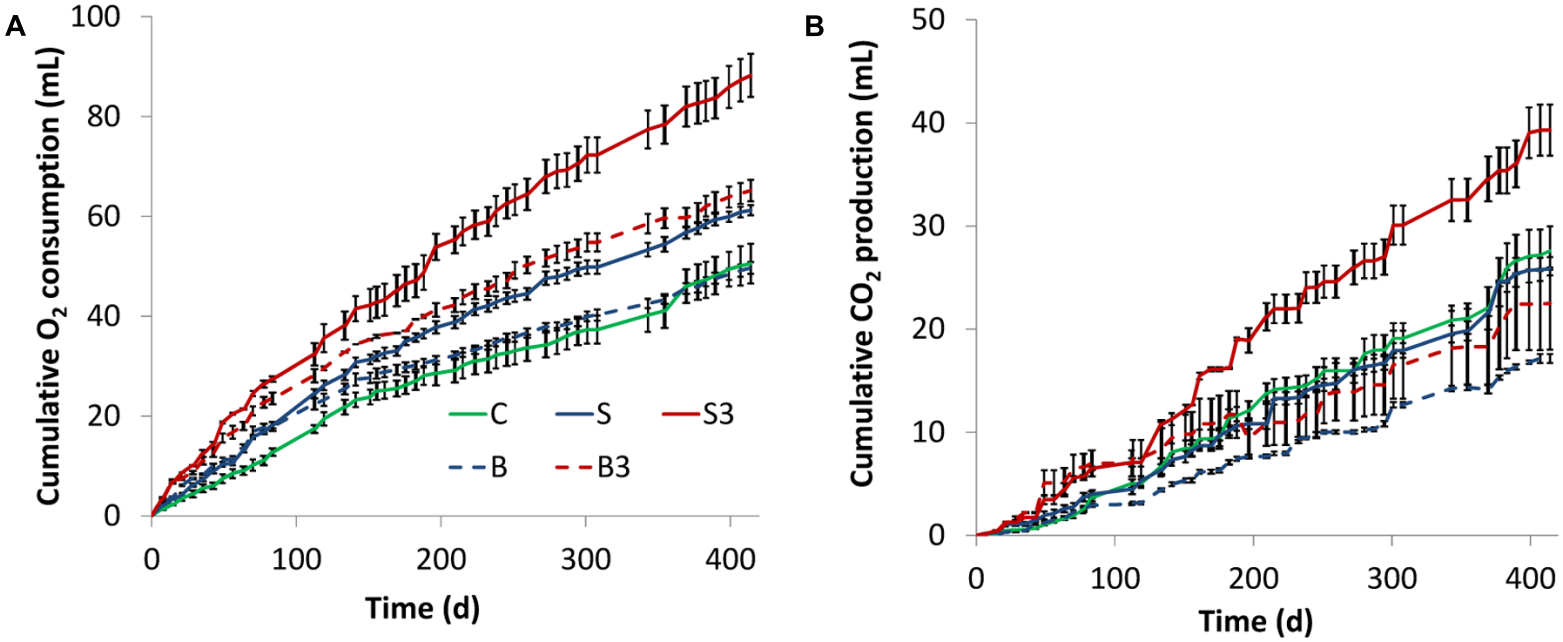
**Cumulative oxygen consumption **(A)** and carbon dioxide evolution **(B)** in the different treatments.** Error bars represent the SE of replicate incubations.

To verify this exciting hypothesis, oxygen consumption and carbon dioxide evolution were monitored, via headspace analysis of the different microcosms, throughout the whole experimental period (**Figure 2**). Live microcosms containing one (treatment “S”) and three (treatment “S^3^”) graphite rods displayed a higher cumulative oxygen consumption compared to the corresponding (live) control microcosms (treatment “C”) lacking the graphite rod(s) (i.e., 61.2 ± 1.0 mL and 88.3 ± 4.3 mL, respectively, versus 50.6 ± 4.0 mL). However, a statistical analysis (two-tailed *t*-test) indicated that only treatment “S^3^” was statistically different (*p* = 0.023) from treatment “C.”

Collectively, these findings indicate that the presence of the graphite electrode(s) within the sediment enhance oxygen uptake from the headspace of the microcosms. Since the graphite rods do not alter the physical–chemical properties of the water–sediment interface and therefore are not expected to modify the rate of oxygen transport from the headspace to the sediment, the observed enhancement of oxygen consumption was likely driven by the electrons released to the graphite rods (from the oxidation of reduced organic and inorganic species in the sediment) which reacted with oxygen forming water as a byproduct.

Unexpectedly, a substantial respiratory activity was observed also in autoclaved controls (treatments “B” and “B^3^”; **Figure 2A**), suggesting the occurrence of non-biological (abiotic) oxidation reactions in the sediment driven by the graphite rod(s) and/or the presence of microorganisms resistant to thermal treatment.

In all incubations, the rate of oxygen consumption (i.e., the slope of the oxygen consumption curve) steadily decreased over time possibly suggesting a gradual loss of biological and/or electrochemical activity of the systems under consideration.

**Figure 2B** shows the cumulative production of carbon dioxide in the different microcosms. In agreement with oxygen consumption data, at the end of the incubation the highest CO_2_ production was observed in the treatment “S^3^” (1.4-fold higher than in treatment “C,” *p* = 0.04 based on two-tailed *t*-test), whereas a lower (and statistically indistinguishable, *p* = 0.49) production was observed in treatments “S” and “C” (**Figure 2B**). Notably, a substantial CO_2_ production was also observed in autoclaved treatments (i.e., “B^3^,” “B”), hence proving an additional line of evidence of the inefficiency of the thermal treatment for microcosm sterilization. Finally, throughout the incubation negligible methane formation was observed in all treatments.

### Sulfate Consumption

At fixed times (i.e., day 0; day 200; day 417), one bottle from each treatment was sacrificed and the liquid phase was analyzed by IC for determination of seawater anions, with main attention being paid at sulfate which is a key respiratory electron acceptor in marine environments.

**FIGURE 3:**
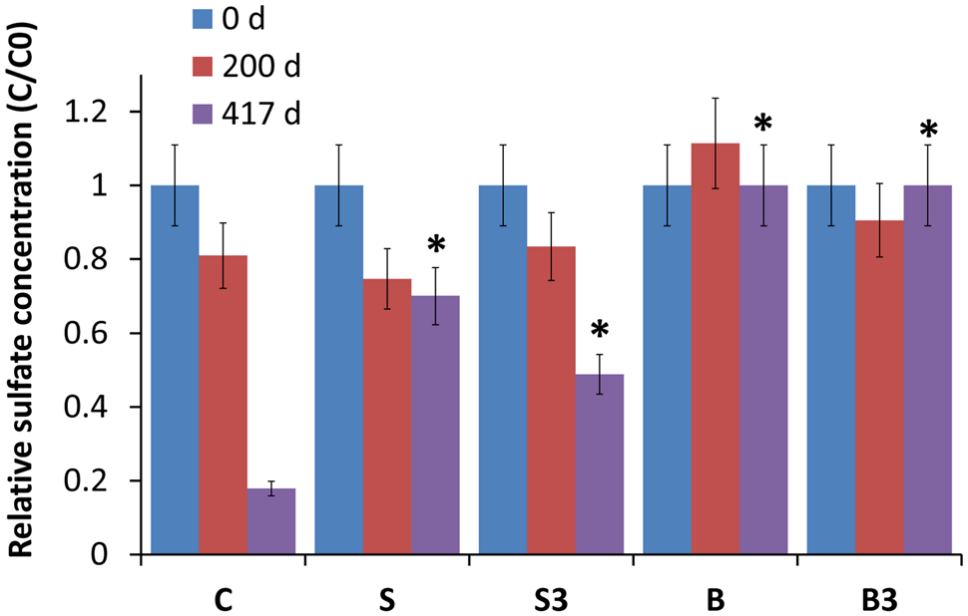
**Relative sulfate concentration in the different treatments, measured after 0, 200, and 417 days of incubation.** Error bars represent the SE of replicate samples. Statistically significant differences (with respect to the corresponding values of treatment “C”) are noted at the ^∗^*p* = 0.05 thresholds from two-tailed *t*-test.

Analyses revealed the occurrence of sulfate reduction in all the biotic treatments (“C,” “S^3^,” “S”), but not in the autoclaved ones (“B” and “B^3^”; **Figure 3**). Notably, sulfate reduction in treatment “C” was substantially higher (*p* < 0.05; two-tailed *t*-test) than in the “snorkels,” with 82 ± 9% of sulfate removal in 417 days compared with 51 ± 6 and 30 ± 3% of sulfate removal in treatment “S^3^” and treatment “S,” respectively. On day 417, the observed difference between treatment “S” and treatment “S^3^” was found to be not statistically significant (*p* = 0.16, two-tailed *t*-test).

### Total Petroleum Hydrocarbons (TPH) Degradation

On day 0, 200, and 417, the different treatments were also analyzed to determine (by GC-FID following accelerated solvent extraction with an acetone:hexane mixture) the concentration of TPH in the sediments. The initial (day 0) concentration of TPH in the different microcosms was the following (in mg/g of dry sediment): treatment “C” = 12.3 ± 0.20; treatment “S” = 11.8 ± 1.01; treatment “S^3^” = 11.9 ± 0.12; treatment “B” = 12.1 ± 0.14; treatment “B^3^” = 11.3 ± 0.11.

**FIGURE 4:**
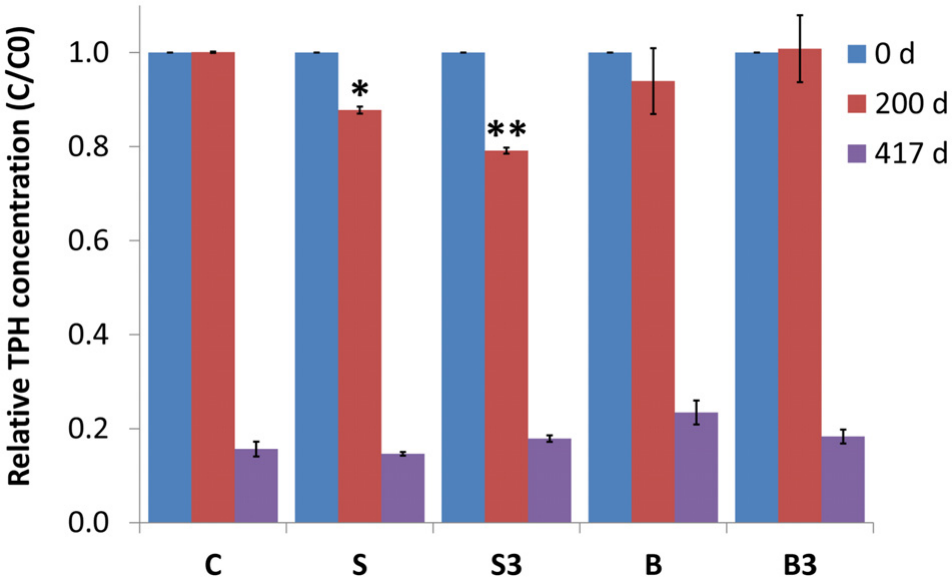
**Relative total petroleum hydrocarbon (TPH) concentration (C/C0) in the different treatments measured after 0, 200, and 417 days of incubation.** Error bars represent the SE of replicate samples. Statistically significant differences (with respect to the corresponding values of treatment “C”) are noted at the ^∗^*p* = 0.05 and ^∗∗^*p* = 0.01 thresholds from two-tailed *t*-test.

After 200 days of incubation, while a negligible degradation of TPH was noticed in snorkel-free biotic and autoclaved controls, a significant reduction of 12 ± 1% (*p* = 0.004) and 21 ± 1% (*p* = 0.001) was observed in microcosms containing one and three snorkels, respectively (**Figure 4**). This important finding provides a straightforward confirmation of the effectiveness of the proposed approach to accelerate hydrocarbons biodegradation in contaminated sediments.

Following a more prolonged incubation (day 417), an extensive degradation of TPH occurred in all treatments, including the autoclaved controls (i.e., treatment “B” and “B^3^”), with removals exceeding 80% in most treatments (**Figure 4**). In correspondence to this last sampling time, the beneficial effect of the “snorkels” was no longer evident (**Figure 4**).

Most probably, over time the rate of microbiological reactions taking place in the bulk of the sediment, driven by sulfate and/or the oxygen steadily dissolving from the headspace of the bottles, exceeded the rate of electrode-driven reactions. This was likely due to a number of factors such as the relatively low surface area of electrodes compared to the amount of sediment used and the batchwise type of incubation which limited the possibility to consistently maintain a spatial separation between the oxic and the anoxic zones. On the other hand, it cannot be excluded that the rate of electrodic reactions also decreased over time due to a gradual passivation of the electrodic surfaces induced by deposition of non-conductive minerals such as sulfides.

### Microbiological Characterization by CARD-FISH

**FIGURE 5:**
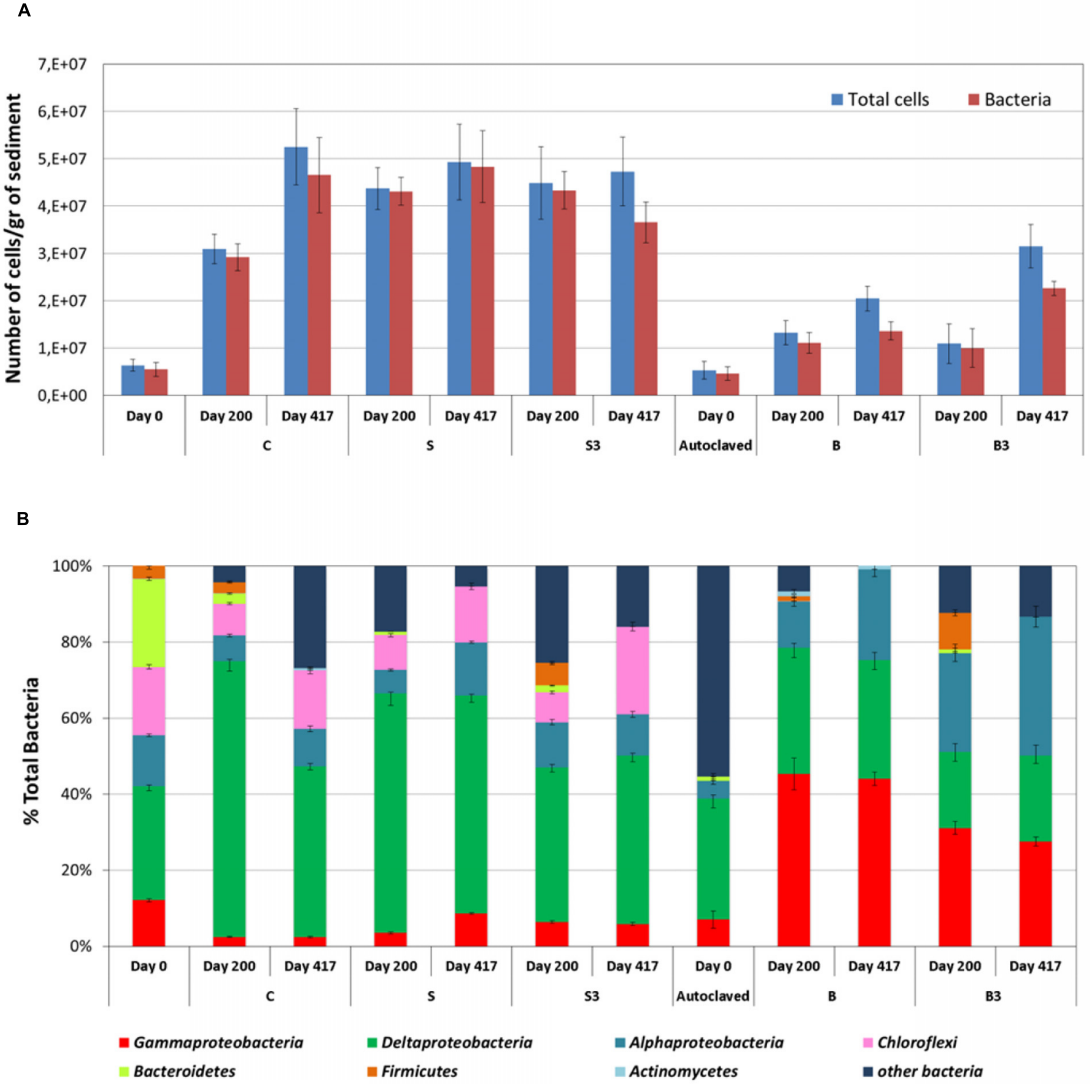
**(A)** Amount of total cells and Bacteria in the sediment from the different treatments as measured after 0, 200, and 417 days of incubation. **(B)** Analysis of the bacterial component of the microbial community of the sediment using group-specific Catalyzed Reporter Deposition-Fluorescence *In Situ* Hybridization (CARD-FISH) probes (samples taken at the end of the incubation). Error bars represent the SE of replicate samples.

A whole-cell detection method was applied for the characterization of the microbial communities growing in the sediment and at the surface of the graphite rods (i.e., “the snorkels”) in the different microcosms. As shown in **Figure 5A**, near the totality of the microbial population in the sediment was composed of Bacteria, with a ratio Bacteria/total DAPI stained cells ranging between 83 and 99%. The abundance of total microbial cells and Bacteria in the sediment samples from the live control (treatment “C”) and from the microcosms containing the “snorkel(s)” (treatment “S” and “S^3^”) increased over time, ultimately reaching a similar value of around 5 × 10^7^ cells per gram of sediment at the end of the incubation (i.e., day 417; **Figure 5A**). A statistically relevant difference among the treatments was observed on day 200 whereby the concentration of total cells in the microcosms containing the snorkel(s) was higher than in the corresponding control (4.4 ± 0.4 × 10^7^ and 4.5 ± 0.4 × 10^7^ cells per gram of sediment in treatment “S” and “S^3^,” respectively, versus 3.1 ± 0.3 × 10^7^ cells per gram of sediment in treatment “C”; *p* < 0.001 for both “S” and “S^3^” treatments, two-tailed *t*-test; **Figure 5A**).

In accordance with oxygen consumption, CO_2_ evolution, and TPH degradation data, an increase in total cells and Bacteria was observed also in the autoclaved controls (treatment “B” and “B^3^”) confirming the occurrence of growth of microorganisms resistant to the thermal sterilization treatment. However, the total cell concentration was substantially lower than in the non-autoclaved treatments (*p* < 0.001, two-tailed *t*-test; **Figure 5A**). This finding is in line with previous studies which showed microbial activity in sediment samples also after several serial sterilization treatments (O’Sullivan et al., 2015). Consistent with the lack of methanogenic activity in the microcosms, no archaeal cells were detected by CARD-FISH.

The structure of the sediment biomass was further detailed by using group specific probes (**Figure 5B**). *Deltaproteobacteria* were the main component in the live microcosms (“C,” “S,” and “S^3^”), accounting from 44 to 57% of total Bacteria at the end of the incubation (**Figure 5B**). Since many sulfate-reducing bacteria (SRB) are affiliated to *deltaproteobacteria*, this finding is consistent with the occurrence of active sulfate reduction in the live microcosms. SRBs include a variety of different families like *Desulfovibrionaceae, Desulfobacteriaceae*, and *Syntrophobacteraceae* often found responsible of oil degradation in contaminated sediments (Miyatake et al., 2009; Sherry et al., 2013; Cravo-Laureau and Duran, 2014; Genovese et al., 2014).

Differently from live microcosms, *gammaproteobacteria* (up to 45.3% of total bacteria) and *alphaproteobacteria* (up to 53%) were the main bacterial components in the autoclaved treatments “B” and “B^3^.” Several aerobic marine hydrocarbon-degrading bacteria, affiliated to *gammaproteobacteria* have been described in the literature (Yakimov et al., 2007). Accordingly, the extensive TPH degradation observed in autoclaved treatments (at least after 417 days of incubation) can probably be attributed to these microorganisms, which likely used the oxygen slowing diffusing into the sediment from the headspace of the microcosms.

*Deltaproteobacteria* were the main bacterial component soon after the autoclaving together with a large portion of bacteria not identified with the group specific probes applied in this study. Autoclave-surviving SRB were recently described in sediment slurries due to the widespread occurrence of bacterial spores in marine sediment (O’Sullivan et al., 2015). The thermal treatment negatively impacted on the survival of some of the groups originally present in the untreated sediment (to) such as *Chloroflexi* and *Bacteroidetes* which almost disappeared in the sediment after autoclaving (**Figure 5B**).

The biofilm growing attached to the surface of the graphite rods (the segment buried within the sediment) also primarily consisted of Bacteria. Overall, the total amount of cells detached from the surface of the graphite rods in treatment “S” and “S^3^” was over four-times higher (*p* < 0.05, two-tailed *t*-test) than that detached from the graphite rods in the autoclaved treatments “B” and “B^3^” (e.g., 8.0 × 10^7^ cells from treatment “S” vs. 2.0 × 10^7^ cells from treatment “B”).

**FIGURE 6:**
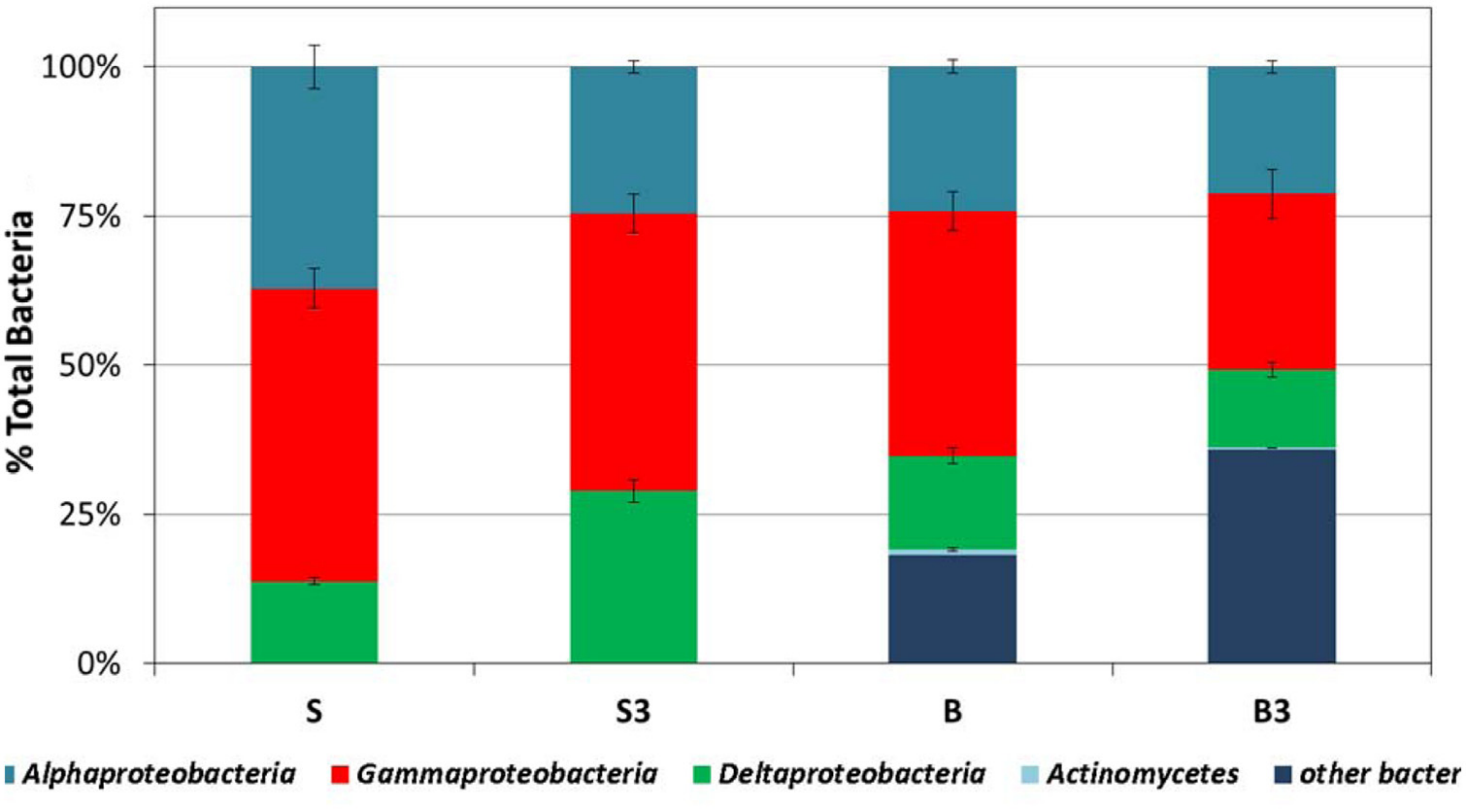
**Analysis of the bacterial component of the microbial community attached at the surface of the graphite rods, using group-specific CARD-FISH probes (samples taken at the end of the incubation).** Error bars represent the SE of replicate samples.

At the end of the incubation, in all treatments the biofilm primarily consisted of *alphaproteobacteria* (accounting for around 25–35% of total Bacteria), *gammaproteobacteria* (accounting for around 30–50% of total Bacteria) and, although to a minor extent, *deltaproteobacteria* (accounting for around 15–30% of total Bacteria; **Figure 6**). In the autoclaved treatments (“B” and “B^3^”) a significant fraction (up to 35%) of total Bacteria remained unidentified (**Figure 6**). Interestingly, the predominance of *alphaproteobacteria* and *gammaproteobacteria* at the surface of the graphite rods suggests the existence of an ecological niche for these microorganisms. A wide diversity of chemoautotrophic *alphaproteobacteria* and *gammaproteobacteria* responsible for sulfur oxidation in marine sedimentary environments have been described in the literature (Meyer and Kuever, 2007; Meyer et al., 2007; Yamamoto and Takai, 2011; Lenk et al., 2012), hence raising the intriguing possibility that these microorganisms were responsible for the bioelectrochemical oxidation of sulfur, deriving from the abiotic electrochemical oxidation of sulfide, to sulfate with the graphite electrode serving as electron acceptor.

Notably, the capacity to oxidize sulfur to sulfate with an electrode serving as direct electron acceptor has been documented also in *deltaproteobacteria*, the other bacterial component identified at the surface of the graphite rods, such as *Desulfuromonas* strain TZ1 (Zhang et al., 2014). Furthermore, it should also be noted that the vast majority of Bacteria isolated so far with the capability to link the anaerobic oxidation of organic substrates to electrode reduction, such as *Geobacter, Shewanella, Geoalkalibacter*, also belong to the subclass of *deltaproteobacteria* (Bond and Lovley, 2003; Biffinger et al., 2011; Badalamenti et al., 2013). Hence, it cannot be excluded that their occurrence at the surface of the graphite rods can be associated to the direct oxidation of TPH and possibly other organic substrates contained in the sediment, with the electrode serving as direct electron acceptor. In spite of these considerations, the identity of the electrode-associated microbes needs to be further elucidated and efforts are certainly needed to unravel their specific function and electron transfer strategies.

### Surface Characterization of the “Snorkels” by XPS

X-ray photoelectron spectroscopy was employed to identify the chemical species present on the surface of the graphite rods retrieved, at the end of the incubation, from the treatments “S” and “B.” For comparative purposes, an identical (yet unused) graphite rod was also analyzed. Besides the trace amounts of impurities, mainly consisting of silicon (as SiO_2_), oxygen (as OH^-^), and chlorine (as Cl^-^), photoemission spectra of the untreated graphite rod revealed only the presence of graphitic carbon (**Table 1**). The C 1s spectrum of carbon in the autoclaved treatment “B” was very similar to that of unused graphite, showing the presence of graphitic carbon only.

**Table 1:** Results of X-ray photoelectron spectroscopy (XPS) analysis of untreated graphite rod and snorkels from the treatments “S” and “B.”

	Carbon (1s)		Oxygen (1s)	Nitrogen (1s)		Sulfur (2p)		Iron (2p)
Chemical bond	Graphitic	C = O, C-N	Oxides	C = NH C-NH_2_	NH_3_ NH_2_OH	Sulfide	Sulfate	Fe^3+^
Untreated graphite rod (atomic %)	100.0	–	–	–	–	–	–	–
Snorkel treatment “S” (atomic %)	68.0	7.4	15.5	3.3	2.9	0.2	0.6	2.1
Snorkel treatment “B” (atomic %)	98.3	–	–	–	1.3	0.1	0.3	–

In contrast, the C 1s spectrum of the graphite rod deriving from the treatment “S” clearly revealed the presence of the second component at 287.7 eV due to the bonds of C = O and/or C-N, most likely attributable to the presence of adherent bacterial cells. Consistently, the nitrogen spectrum revealed the presence of N 1s peak with binding energy of 399.0 eV which is characteristic for C = NH, C-NH_2_ bonds. Even though microbiological and chemical analyses indicated the presence of bacteria also in the autoclaved treatment “B,” they could not be detected by XPS probably due to their much lower abundance compared to the treatment “S.” As far as the sulfur spectrum S 2p is concerned, XPS analyses did not reveal the presence of elemental sulfur but only sulfides (about 164 eV) and sulfates (about 169 eV; Gusmano et al., 2004), particularly after the treatment “S,” hence providing an additional line of evidence of the involvement of the “Snorkel” in the redox cycling of sulfur species.

Another interesting result of the XPS analysis is the relatively high abundance (2.1 atomic %) of iron ions Fe^3+^ (710.2 eV; Mari et al., 2009) on the surface of graphite rods deriving from the treatment “S,” whereas it was undetectable in the other samples. The presence of iron was also visually confirmed by the fact that when the graphite rod from the treatment “S” was removed from the serum bottle and briefly exposed to the atmospheric oxygen before introducing into XPS apparatus, its surface immediately turned reddish. Interestingly, the reddish color is typical of “electro-active” biofilm harboring high amounts of Fe-bearing *c*-type cytochromes which are key redox protein involved in the electron transfer processes between the microbial cells and the electrodes.

## Discussion

**FIGURE 7:**
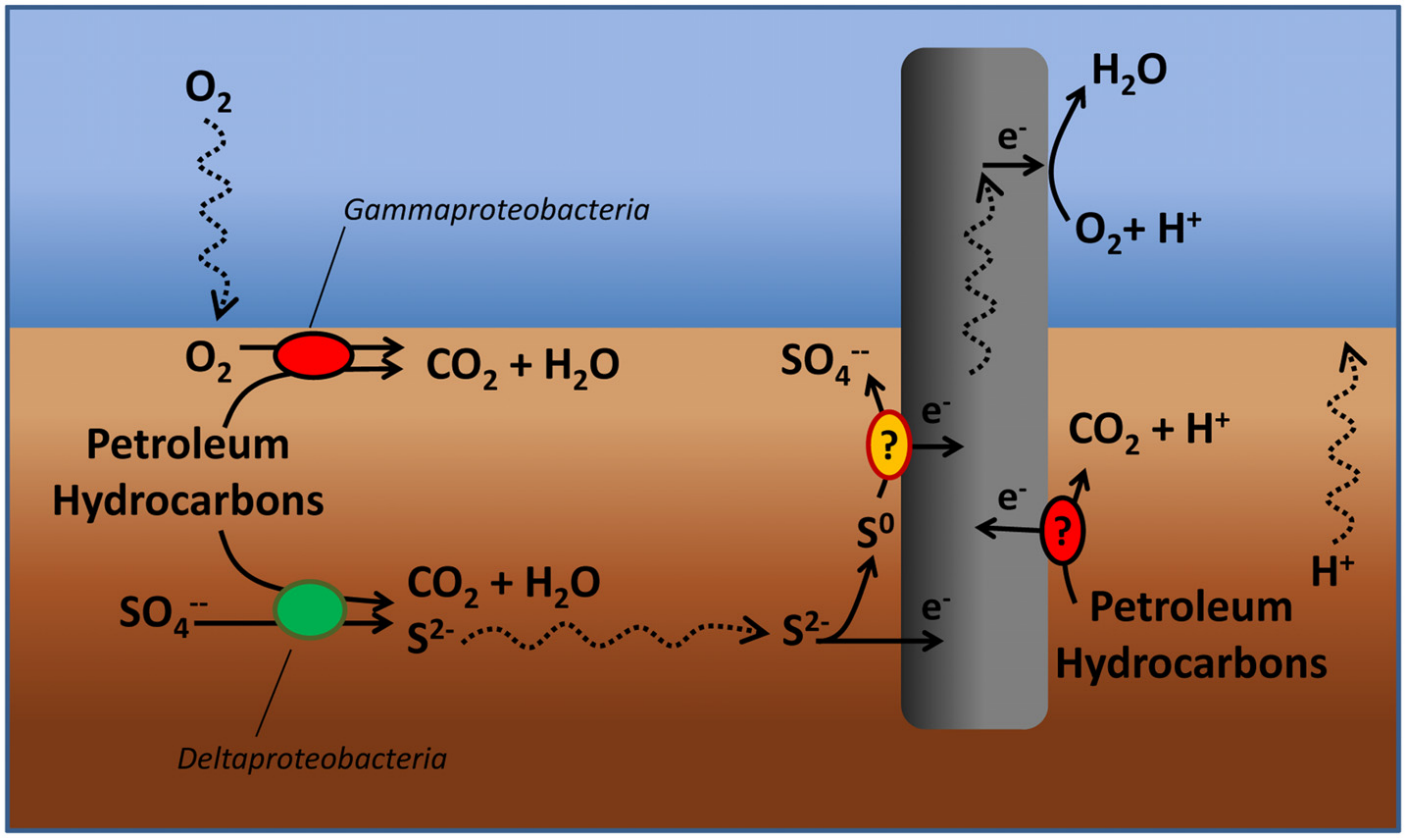
**Tentative model depicting the effects of the “snorkel” on chemical and microbiological reactions taking place in a petroleum hydrocarbon contaminated sediment**.

This study presented the proof of concept of the “Oil-Spill Snorkel,” a novel bioelectrochemical approach to accelerate *in situ* the biodegradation of petroleum hydrocarbons in anoxic marine sediments. Collectively, the results of this study which are based on a comprehensive set of chemical, microbiological, and spectroscopic data have pointed out that the introduction of a conductive graphite rod in the sediment allows creating a bioelectrochemical connection between two spatially separated redox zones (i.e., the anoxic sediment and the overlying oxic water) and, by so doing, affecting a number of microbiological and chemical reactions occurring within the sediment (**Figure 7**). The snorkel accelerated the rate of oxidative reactions taking place within the sediment, as documented by the enhancement of the rate of oxygen uptake and carbon dioxide evolution. Consistently, also the initial rate of petroleum hydrocarbons biodegradation was substantially increased, even though upon extended incubation (over 400 days) the final hydrocarbons removal was similar among treatments. Probably, a greater beneficial effect of the snorkel could be obtained in larger-scale and continuous-flow systems in which the spatial separation between the oxic and anoxic redox zones can be stably maintained over time.

A striking effect of the snorkel was also noticed on sulfate reduction which proceeded at slower rates in treatments containing the electrode(s). A possible explanation for this finding is the preferential use of the “snorkel” over sulfate as respiratory electron acceptor for the oxidation of organic substrates in the sediment. In principle, this hypothesis is consistent with the higher redox potential of oxygen (E°’ O_2_/H_2_O = +0.82 V), which is the ultimate sink for electrons released to the snorkel, compared to sulfate (E°’ SO_4_^2-^/HS^-^ = –0.217 V).

On the other hand, another possible explanation is that the “snorkels” facilitate the back-oxidation (to sulfate) of the sulfide generated in the sediment from the activity of sulfate-reducing microorganisms, hence resulting in an apparently lower sulfate reduction. However, under abiotic (purely electrochemical) conditions sulfide is only partially oxidized to elemental sulfur (Rabaey et al., 2006; Dutta et al., 2008; Sun et al., 2009); hence, the complete conversion of sulfide to sulfate requires the initial abiotic step (from sulfide to elemental sulfur) to be followed by the microbiological oxidation of elemental sulfur to sulfate (Gong et al., 2013). Recently, a number of microorganisms such as *Desulfobulbus propionicus* (Holmes et al., 2004a,b) and *Desulfuromonas* strain TZ1(Zhang et al., 2014) with the capability to oxidize elemental sulfur using a graphite electrode as direct electron acceptor have been described in the literature.

At present, it is unclear whether the effect of the snorkel on hydrocarbons biodegradation was direct (e.g., the graphite electrode served as a direct electron acceptor for hydrocarbons oxidation), indirect (e.g., the electrode somehow stimulated the activity of hydrocarbon-oxidizing sulfate-reducing bacteria) or both. To specifically address this issue, future research efforts will have to focus on the identification of involved biochemical pathways of hydrocarbons activation and biodegradation, through for instance the detection of signature metabolites.

An interesting finding of the study was the formation on the surface of the snorkels of a Fe^3+^-rich, reddish biofilm dominated (among the others) by *deltaproteobacteria* which could be an indirect indication that the biofilm was capable to engage in extracellular electron transfer processes with the electrode. More specific (bio)electrochemical investigations are, however, needed to confirm this intriguing hypothesis.

From an applicative standpoint, the oil-spill snorkel could potentially represent a groundbreaking *in situ* alternative to more expensive remediation options relying on dredging or on often ineffective practices of oxygen delivery to the sediment. The oil-spill snorkel is indeed a fully passive bioelectrochemical system which, upon installation does not necessitate the input of external energy and requires little to no maintenance, employing corrosion-resistant materials (i.e., carbon based electrodes) suitable for use in marine environments. Also compared to other bioelectrochemical systems previously applied to the treatment of petroleum hydrocarbons in contaminated soil and sediments, the oil-spill snorkel has several potential advantages such as the fact that it does not necessitate the presence of expensive electrode materials and catalysts as well as the use of membranes, thereby suggesting a greater potential for practical application (Morris and Jin, 2012; Lu et al., 2014a,b).

However, a number of factors possibly influencing its long-term operation still need to be evaluated, including the possible formation of non-conductive precipitates (e.g., sulfides) on the surface of the snorkel which may eventually lead to a gradual passivation of the electrode.

Clearly, it is important to mention that the geometry and setup of the electrodes employed in this study may not reflect the optimal configuration for full-scale “real world” applications. Most probably, in order to treat extended surface, a horizontal positioning of the electrodes (rather than the vertical one used in this study) would be preferable. Furthermore, crucial to the scaling up of the technology is also the radius of influence of the snorkel which will require an experimental study at a larger scale to be precisely identified.

## Conflict of Interest Statement

The authors declare that the research was conducted in the absence of any commercial or financial relationships that could be construed as a potential conflict of interest.

## Footnotes

1 http://www.biomers.net

2 http://www.microbial-ecology.net/probebase/
